# Atypical Presentation of Hepatocellular Carcinoma Mimicking a Gastric Hepatoid Adenocarcinoma: A Case Report

**DOI:** 10.1097/MD.0000000000001101

**Published:** 2015-07-13

**Authors:** Aymeric Becq, Christian Mateescu, David Khayat, Mohamed Bouattour

**Affiliations:** From the Department of Medical Oncology (AB, CM, MB), Beaujon University Hospital (AP-HP – PRES Paris 7 Diderot), Clichy; Department of Medical Oncology (CM, DK), La Pitié Salpêtrière University Hospital (AP-HP – PRES Paris 6 Pierre et Marie Curie), Paris; and Department of Hepatology (MB), Beaujon University Hospital (AP-HP – PRES Paris 7 Diderot), Clichy, France.

## Abstract

The diagnosis of hepatocellular carcinoma (HCC) relies on imaging tools and biopsy. It usually does not present to be a challenge.

Here we report the case of a 69-year-old patient with HCC, initially mistaken for a gastric hepatoid adenocarcinoma (HAC), with a favorable outcome after neoadjuvant chemotherapy.

The initial presentation (clinical signs, morphological features, and histological findings) led to the diagnosis of a gastric hepatoid adenocarcinoma. Neoadjuvant chemotherapy by epirubicin, oxaliplatin, and capecitabine protocol was administered. Biological (alpha-fetoprotein [AFP] decreased by a factor of 10), radiological (−35% RECIST), and histological (20% of necrosis) responses were observed. Complete surgical resection was then performed. The final pathological diagnosis was a well-differentiated HCC, staged pT4 N0 (0/24) R0.

There are no guidelines as to how such tumors should be managed. Nonetheless, neoadjuvant chemotherapy yielded a good outcome. This observation stresses the importance of the final pathological findings and addresses the issue of neoadjuvant therapy in some cases of HCC.

## INTRODUCTION

Hepatocellular carcinoma (HCC) accounts for over 90% of all common liver primary cancers. It often occurs in the setting of an underlying liver disease.^1,2^ Diagnosis is usually based on noninvasive imaging techniques showing characteristic morphological features.^1,2^ Biopsy for pathology analysis is rarely required for diagnosis purposes. Prognosis of HCC depends on the initial tumor staging, which subsequently determines the therapeutic management.^1,2^

Clinicians may be confronted with differential diagnosis including benign and malignant lesions. For instance, hepatoid adenocarcinoma (HAC) is a rare variant of extrahepatic adenocarcinoma that frequently originates from the stomach. The distinction between HCC and HAC may be challenging, because of their similar clinical and pathological features.^3^ Alpha-fetoprotein (AFP) plasma level is often misleadingly elevated,^4^ especially in the setting underlying liver disease such as cirrhosis. Furthermore, histological assessment of HAC specimen shows similar morphological features to those of HCC.^3^ Immunohistochemical staining may be helpful for the diagnosis of HAC showing positive rates of AFP, Hep Par 1, and carcinoembryonic antigen (CEA) stains of 91.6%, 38.1%, and 78.7%, respectively.^3^ The prognosis of HAC is poor, with a life expectancy shorter than that of HCC.^5^

Herein, we report the case of an unusual presentation of HCC, located in the left lobe of the liver and extending to the anterior gastric wall. The tumor was initially mistaken for a gastric HAC. The patient received neoadjuvant chemotherapy followed by radical surgery yielding a prolonged survival.

## CLINICAL CASE

A 69-year-old male presented in February 2012 with epigastric pain, nausea, and melena. He had been treated with nonsteroidal anti-inflammatory drugs (NSAIDS) for the pain. Past medical history included hypertension treated with FORZAAR (hydrochlorothiazide and losartan) and ALPRESS (prazosin hydrochloride). There was no family history, allergies, and alcohol or tobacco intoxication. Clinical findings were a good general condition (Performance Status Score: 0), no lymph nodes, ascites, or other signs of portal hypertension. The body mass index was 28 kg/m^2^.

**FIGURE 1:**
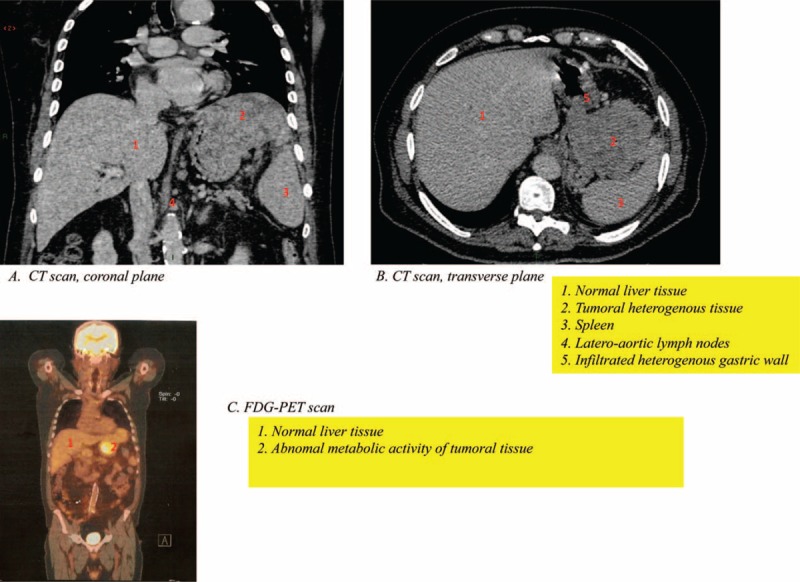
Imaging at diagnosis showing at CT scan (A and B) a large heterogeneous tumor measuring 10 × 8 mm initially the origin was taken for a gastric lesion. The tumor extends to the diaphragm, the splenic hilum, and lymph nodes. The liver seems to be normal with no evident lesions. The positron emission tomography scan showed abnormal metabolic activity located near the liver and the spleen hilum (C). CT = computed tomography.

An upper gastrointestinal endoscopy was performed given the digestive symptoms and showed a 4 to 5-cm-long lesion located in the anterior part of the greater curvature of the stomach. This lesion looked necrotic and bled easily upon contact. Multiple biopsies were performed, and analysis showed an unusual aspect, made of large cells, with an eosinophilic cytoplasm, lacking mucosecretion, presenting a moderate anisokaryosis and many mitoses. These findings were consistent with a gastric HAC. Immunohistochemical analysis showed that the tumor was negative for CK7, CK20, antihepatocyte antibody, chromogranin synaptophysin, CD56, and positive for pankeratin (confirming the epithelial nature) and Ki67 (30%–40%). A computed tomography (CT) scan was performed to evaluate tumor extension and showed a large gastric tumor measuring 10 × 8 mm, extending to the diaphragm, the splenic hilum, and lymph nodes in the lateroaortic region (Figure 1). There were no metastatic lesions (liver or lungs). A positron emission tomography scan showed abnormal metabolic activity located near the liver and the spleen hilum (Figure 1). Endoscopic ultrasonography (US) showed a heterogeneous, hypoechoic tumor, infiltrating the gastric wall as far as the muscularis mucosae. The cardia was tumor free. An infiltration of the left liver lobe, the retroperitoneum, and the spleen were also identified.

AFP serum level was 43.000 UI/mL. CA19.9 and ACE serum levels were within the normal range (15 U/mL and 2 ng/mL, respectively). Liver function tests were unremarkable.

A multidisciplinary consultation meeting was held, and neoadjuvant chemotherapy by epirubicin, oxaliplatin, and capecitabine (EOX) protocol was decided. The patient was informed and gave his oral consent for the treatment strategy. The aim was to perform surgery afterward provided the response be sufficient. Treatment consisted of epirubicin 50 mg/m^2^ (day 1), oxaliplatin 130 mg/m^2^ (day 1), and capecitabine 625 mg/m^2^ b.i.d (from day 1 to day 21). Tolerance was good overall, except an episode of diarrhea (grade 1) and thrombocytopenia (grade 2). The latter led to a 25% reduction of the capecitabine dosage after the first 2 cycles.

**FIGURE 2:**
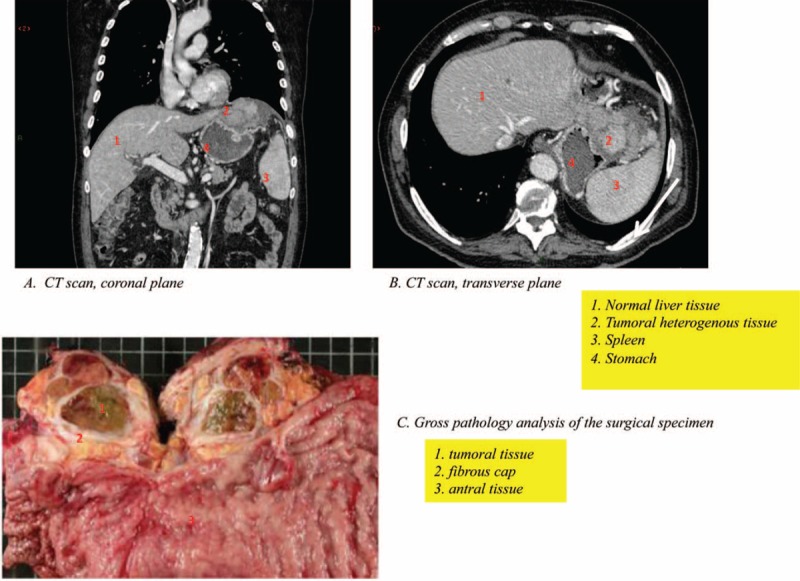
A CT scan performed after 3 cycles of chemotherapy (EOX protocol) showing a partial response with tumor shrinkage (A and B) and gross pathology of surgical specimen showing the macroscopic features of the resected lesion (C). CT = computed tomography, EOX = epirubicin, oxaliplatin, and capecitabine.

The response to chemotherapy was evaluated by CT scan after 3 cycles of chemotherapy, which showed a moderate response, based on RESIST criteria, with a size diminution of 35%. The residual tumor was located in the splenic hilum, the diaphragm, and the left liver lobe (Figure 2). No lesions appeared. AFP serum levels had dramatically decreased to 3.441 UI/mL.

The patient was operated after the fourth cycle of chemotherapy. Gastrectomy with oesojejunal anastomosis, splenectomy, left hepatectomy, and partial phrenicotomy was performed by laparotomy. Macroscopically, the resection was judged complete (R0).

Pathology analysis of the surgical specimen (Figure 2) turned out to be consistent with well-differentiated HCC, originating from the tip of the left liver lobe, infiltrating adjacent organs such as the splenic hilum, the diaphragm, and the gastric wall. The microscopic study showed a lobulated partially necrotic (20%) carcinoma, comprising well-differentiated bile producing hepatoid cells, 70% of which had steatosis. The stroma was abundant and formed hyaline fibrous bands where Mallory corps could be found. Additionally, infiltration of the anterior gastric wall was observed though it did not reach the submucosa. In the surrounding gastric mucosa, a hyperplasic polyp could be seen, and no dysplastic lesion was identified. The remaining gastric mucosa presented signs of chronic atrophic gastritis with lymphoid hyperplasia, but no intestinal metaplasia or dysplasia. The underlying nontumor liver tissue analysis showed discrete fibrosis (F1) and steatosis (<20%).

The resection was judged complete, and the final tumor staging was pT4 N0 (0/24) R0. From June 2012 to December 2013, the patient was under simple surveillance. In December 2013, he presented a pleural effusion and increased AFP serum levels. Imaging did not show macroscopically detectable lesions at that time. A pleuroscopy and upper gastrointestinal endoscopy with biopsies were performed, showing no signs of recurrence of the malignancy. In spite of that, the context, the rising AFP, and the otherwise unexplained pleural effusion lead to the conclusion of an HCC recurrence, with a time to progression after surgery of 19 months. Chemotherapy by EOX protocol was initiated in March 2014, 2 years after the initial diagnosis. The patient died of uncontrolled sepsis in September 2014. At that time, he was still treated by EOX protocol, and recent CT scan had shown stability based on RESIST criteria. Postsurgery survival was of 31 months.

## DISCUSSION

Here we report a patient with an unusual presentation of advanced HCC, most of the tumor being extrahepatic, to the point where the first CT scan had not shown liver-localized tumor. The metastatic extension was also uncommon (splenic hilum, diaphragm, stomach). The absence of underlying liver disease and signs of cirrhosis added to the rare presentation. Furthermore, initial histological findings were consistent with gastric HAC. In most cases, HCC develops in the setting of cirrhosis (>85%). HCC may also occur in the setting of a chronic liver disease but rarely on a normal liver.^1,2^ Diagnosis of HCC is usually based on imaging techniques such as contrast US, MRI, and CT scan showing typical morphological features.^4^ Pathology analysis of biopsy or resected specimen may be useful especially when the diagnosis is difficult, for instance in the case of an atypical clinical and/or imaging presentation.^1,2^ Differential diagnosis includes benign lesions and other malignant liver tumors such as metastatic lesions and cholangiocarcinoma.

In our case, pathology assessment was challenging. A gastric HAC was diagnosed upon initial assessment of multiple biopsies. Few cases of gastric HAC had been reported so far.^3^ Differential diagnosis with HCC may be complicated, given their similarity in terms of histological presentation.^3^ Immunohistochemistry staining provides specific findings that may be helpful in this setting.^3^ Positive AFP and CEA stains are found in the majority of HACs (91.6% and 78.7%). AFP is a well-known tumor marker of HCC. Positive AFP stain is found in 8% to 37% of HCCs that may cause confusion. CEA stain may also be positive in HCC. Hep Par 1 (hepatocyte paraffin 1) is a highly specific marker of HCC, but some gastric HACs may present with positive staining (38.1%).^3^

In terms of epithelial markers, all gastric HAC are positive for CK18 and CK19 stains (100%), and most are positive for AE1 and AE3 (92.3%).^3^ The positive rate of CK19 in HCC is very low and confers a poor prognosis.^6^

All in all, there is no single marker able to differentiate gastric HAC apparently from HCC. CK19, Hep-Par 1, and CEA are nonetheless essential to establish a diagnosis.^3^ The study by Su et al^3^ suggests that new immunohistochemistry markers may be useful to differentiate HAC from HCC. Previous studies showed that palate, lung, and nasal epithelium carcinoma-associated protein (PLUNC) seem promising to that effect whereas CD10 and CDX2 are equivocal.^7,8^

Neither neoadjuvant chemotherapy nor surgery would have been the first treatment approach if the diagnosis of HCC had initially been made. This HCC was stage C according to the Barcelona Clinic Liver Cancer staging classification. Current guidelines do not recommend surgery or neoadjuvant chemotherapy in this setting. Our patient would probably have been treated with sorafenib. The use of sorafenib at this stage is based on the positive results of a large phase III clinical trials and the supposed chemoresistance of HCC.^1,9,10^

Several conventional chemotherapies have been evaluated in advanced HCC with disappointing results in terms of improvement in overall survival, despite varied response rates.^11^ Recently, a randomized phase III Asian clinical trial compared FOLFOX (fluorouracil, folic acid, oxaliplatin) and doxorubicin; 371 cirrhotic patients (Child A or B) presenting a stage B or C HCC were included. Median overall survival was longer in the FOLFOX-treated group (6.4 vs 4.9 months, *P* = 0.07). The progression-free survival was also longer (2.9 vs 1.7 months, *P* < 0.001). The difference in overall survival was not significant although there was a trend.^12^ A French phase II prospective clinical trial evaluated the efficacy of GEMOX (gemcitabine 1000 mg/m^2^ day 1 and oxaliplatin 100 mg/m^2^ day 2); 34 patients presenting a stage B or C HCC were included. Chemotherapy was administered every 2 weeks until progression or severe toxicity. The response rate was of 18% (95% confidence interval [CI]: 8–34). Disease stabilization rate was 58% (comprising 5 minor responses). Altogether, disease control was achieved in 76% of all patients. Median progression-free survival was 6.3 months (95% CI: 4.3–10.1), and median overall survival was 11.5 months (95% CI: 8.5–14.3).^13^

Our patient had a satisfactory response to chemotherapy, allowing a surgical resection. The chemosensitivity was also an uncommon feature of our case. We believe that chemotherapy could be of interest in highly selected cases of HCC. Unfortunately, there has yet to be markers that could help distinguish these particular tumors. Studies are warranted to identify these markers.

## CONCLUSION

We present an unusual case of a mostly extrahepatic HCC with a challenging diagnosis given histological findings mimicking an HAC. This case highlights that HCC may present in an atypical way and that the differential diagnosis with HAC may be difficult. This case shows the importance of the final histological analysis. This case also suggests that HCC may benefit from conventional chemotherapy. Consequently, among the different subtypes of HCC, some may respond to chemotherapy. New markers are needed to identify tumors susceptible of being sensitive to chemotherapy.

## Acknowledgment

The authors would like to acknowledge “la Foundation *avec*” that funded all costs associated with the publishing of the present manuscript.

## References

[1] EASL-EORTC clinical practice guidelines: management of hepatocellular carcinoma. *J Hepatol* 2012; 56:908–943. 22424438 10.1016/j.jhep.2011.12.001

[2] Bruix J Sherman M American Association for the Study of Liver Diseases Management of hepatocellular carcinoma: an update. *Hepatology* 2011; 53:1020–1022. 21374666 10.1002/hep.24199 PMC3084991

[3] Su JS Chen YT Wang RC Clinicopathological characteristics in the differential diagnosis of hepatoid adenocarcinoma: a literature review. *World J Gastroenterol* 2013; 19:321–327. 23372352 10.3748/wjg.v19.i3.321 PMC3554814

[4] Gomaa AI Khan SA Leen EL Diagnosis of hepatocellular carcinoma. *World J Gastroenterol* 2009; 15:1301–1314. 19294759 10.3748/wjg.15.1301 PMC2658831

[5] Liu X Cheng Y Sheng W Analysis of clinicopathologic features and prognostic factors in hepatoid adenocarcinoma of the stomach. *Am J SurgPathol* 2010; 34:1465–1471. 10.1097/PAS.0b013e3181f0a873 20871221

[6] Lee JI Lee JW Kim JM Prognosis of hepatocellular carcinoma expressing cytokeratin 19: comparison with other liver cancers. *World J Gastroenterol* 2012; 18:4751–4757. 23002345 10.3748/wjg.v18.i34.4751 PMC3442214

[7] Metzgeroth G Ströbel P Baumbusch T Hepatoid adenocarcinoma—review of the literature illustrated by a rare case originating in the peritoneal cavity. *Onkologie* 2010; 33:263–269. 20502062 10.1159/000305717

[8] Sentani K Oue N Sakamoto N Gene expression profiling with microarray and SAGE identifies PLUNC as a marker for hepatoid adenocarcinoma of the stomach. *Modern Pathol* 2008; 21:464–475. 10.1038/modpathol.3801050 18204429

[9] Llovet JM Ricci S Mazzaferro V Sorafenib in advanced hepatocellular carcinoma. *N Engl J Med* 2008; 359:378–390. 18650514 10.1056/NEJMoa0708857

[10] Bouattour M Marijon H Dreyer C [Targeted therapies in hepatocellular carcinoma]. *Presse Med* 2010; 39:753–764. 20378303 10.1016/j.lpm.2009.11.016

[11] Taieb J Barbare JC Rougier P Medical treatments for hepatocellular carcinoma (HCC): what's next? *Ann Oncol* 2006; 10:308–314. 10.1093/annonc/mdl279 17018744

[12] Qin S Bai Y Lim HY Randomized, multicenter, open-label study ofoxaliplatin plus fluorouracil/leucovorin versus doxorubicin as palliative chemotherapy in patients with advanced hepatocellular carcinoma from Asia. *J Clin Oncol* 2013; 31:3501–3508. 23980077 10.1200/JCO.2012.44.5643

[13] Louafi S Boige V Ducreux M Gemcitabine plus oxaliplatin (GEMOX) in patients with advanced hepatocellular carcinoma (HCC): results of a phase II study. *Cancer* 2007; 109:1384–1390. 17330837 10.1002/cncr.22532

